# Dataset on assessment of physical and chemical quality of groundwater in rural drinking water, west Azerbaijan Province in Iran

**DOI:** 10.1016/j.dib.2018.09.078

**Published:** 2018-10-09

**Authors:** Majid Radfard, Hamed Soleimani, Abooalfazl Azhdarpoor, Hossein Faraji, Amir Hossein Mahvi

**Affiliations:** a Research Center for Health Sciences, Institute of Health, Department of Environmental Health, School of Health, Shiraz University of Medical Sciences, Shiraz, Iran; b Department of Environmental Health Engineering, School of Health, Shiraz University of Medical Sciences, Shiraz, Iran; c Department of Environmental Health Engineering, School of Public health, Tehran University of Medical Sciences, Tehran, Iran; d Students Research Committee, Hamadan University of Medical Sciences, Hamadan, Iran; e Center for Solid Waste Research, Institute for Environmental Research, Tehran University of Medical Sciences, Tehran, Iran

**Keywords:** Drinking water, Physico-chemical quality, West azerbaijan province, Iran

## Abstract

Analyzing the quality of drinking water and comparing it with standards, provides useful information regarding in the state of water supply and health protection to consumers. In the current research, the quality of drinking water in the cities of West Azerbaijan province has been investigated. In the current study, the results of drinking water analysis in 17 counties of West Azerbaijan province (except Urmia city), including 355 analyzes were conducted in 2016. The results were analyzed by SPSS software and compared with the national standard. Based on the analysis, the hardness of drinking water in the West Azerbaijan province ranged from 22 to 912 mg/L as calcium carbonate, and the average of the experiment was measured to be 136 ± 327 mg/L as calcium carbonate. The TDS values in this study were 39–1710 mg/L, and on average 397.7 ± 265.8 mg/L. Also, based on the analyzes performed in this study, the Fluoride concentration was from 0 to 3.45 mg/L, and on average 323.376 ± 0.05 mg/L and the Nitrate concentration was 0–218 mg/L and on average 3.58 ± 1.1 mg/L.

**Specifications table**

**Table:** 

Subject area	Chemistry
More specific subject area	Describe narrower subject area
Type of data	Tables, figure
How data was acquired	The studied subject was drinking water resources in the cities of West Azerbaijan province. In order to carry out this study, the results of chemical analysis of urban and rural water resources of 13 cities of West Azerbaijan province were collected as a sample in 2017. In the following, data on the concentration of cations and anions, hardness, electrical conductivity, turbidity, TDS, and alkalinity were extracted and the values of mean, standard deviation and correlation coefficient were calculated
Data format	Raw, analyzed
Experimental factors	The mentioned parameters above, in abstract section, were analyzed according to the standards for water and wastewater treatment handbook.
Experimental features	The levels of physical and chemical parameters were determined.
Data source location	West Azerbaijan province, Iran
Data accessibility	The data are available whit this article

**Value of the data**

• Determination of the physical and chemical parameters including F^−^, ${\mathrm{NO}}_{3}^{-}$ , Cl^−^, ${\mathrm{SO}}_{4}^{2-}$ , K^+^, Na^+^, Mg^2+^, Ca^2+^, pH, ALK, Turbidity, TH, TDS and EC in ground water was investigated the cities of West Azerbaijan province, Iran.
• In a number of cities in the West Azerbaijan province, the results of comparing the quality parameters of drinking water with national and international standards showed that some of these parameters were not within the standard range.
• Fluoridation of drinking water in cities area with less than the WHO optimum value is recommended.
• Based on the data, defluoridation water could be recommended in fluorotic cities area.
• According to the Pearson correlation, there is a positive correlation between the concentration of water fluoride and EC, TH, Na^+^, K^+^, ALK and ${\mathrm{SO}}_{4}^{2-}$.

## 1 Data

See Table 1, Table 2, Table 3.

**Table 1:** Statistical description of quality parameters of drinking water resources of West Azerbaijan province in 2017.

Statistical	F^−^ (mg/L)	${\mathrm{NO}}_{3}^{-}$ (mg/L)	Cl^−^ (mg/L)	${\mathrm{SO}}_{4}^{2-}$ (mg/L)	K^+^ (mg/L)	Na^+^ (mg/L)	Mg^2+^ (mg/L)
Mean	0.32	3.58	48.21	72.26	3.21	39.4	34
SD	0.37	11.72	85.34	103.41	3.67	34.16	33.1
Min	0	0	2	1	0	0	0
Max	3.45	218	700	980	26	162	440
Statistical	Ca^2+^ (mg/L)	pH	ALK (mg/L as CaCO_3_)	Turbidity (NTU)	TH (mg/L as CaCO_3_)	TDS (mg/L)	EC (µs/cm)
Mean	76.1	7.4	283.97	3.47	327.28	39	810.89
SD	33	0.25	117.32	32.73	136.6	265.79	527.29
Min	5	7	0	0	22	39	82
Max	253	8	720	536	912	1710	3360

**Table 2:** Status of public and sanitary parameters of water in the cities of West Azerbaijan province in 2017.

City	Statistical	F^−^ (mg/L)	${\mathrm{NO}}_{3}^{-}$ (mg/L)	pH	ALK (mg/L as CaCO_3_)	TH (mg/L as CaCO3)	EC (µs/cm)
Sardasht	Mean	0.15 ± 0.22	2.43 ± 1.32	7.4 ± 0.267	208.6 ± 64	230.1 ± 78.3	442.2 ± 143.2
	Min	0	1	7	40	56	82
	Max	1	7	8	340	456	829
Mahabad	Mean	0.23 ± 0.23	10 ± 41.5	7.25 ± 0.2	301.3 ± 144.6	354 ± 161	827.7 ± 482
	Min	0	1	7	80	76	260
	Max	0.77	218	8	520	604	1880
Takab	Mean	0.2 ± 0.12	2.6 ± 1.2	5.7 ± 0.2	249.2 ± 77.5	298.2 ± 132	661.1 ± 363
	Min	0	0	7	68	80	151
	Max	0.58	12	8	368	596	1740
Naqadeh	Mean	0.22 ± 0.11	2.7 ± 2.1	7.33 ± 0.26	309 ± 76.1	305.2 ± 50	657.5 ± 135.8
	Min	0.04	1	7	172	200	477
	Max	0.5	10	8	460	428	927
Chaypareh	Mean	0.22 ± 0.11	3.24 ± 2.5	7.33 ± 0.26	313.25 ± 81	291 ± 47	683.6 ± 145.7
	Min	0.04	1	7	172	200	497
	Max	0.5	10	8	460	388	927
Khoy	Mean	0.2 ± 0.22	2.68 ± 2	7.46 ± 0.2	284.7 ± 147	361.1 ± 156.8	861.03 ± 565.2
	Min	0	0	7	72	148	330
	Max	0.9	12	8	704	820	2510
Miandoab	Mean	0.35 ± 0.28	1.93 ± 0.87	7.48 ± 0.22	285.9 ± 80.2	355.7 ± 153	993.6 ± 543.5
	Min	0	1	7	128	164	322
	Max	1.44	4	8	404	828	2920
Oshnavieh	Mean	0.16 ± 0.14	2.52 ± 1.4	7.52 ± 0.3	268.25 ± 136	289.6 ± 114	545.87 ± 310
	Min	0.01	1	7	72	72	149
	Max	0.4	7	8	560	460	1210
Salmas	Mean	0.27 ± 0.23	3.36 ± 2.5	7.3 ± 0.13	311 ± 73.1	380.2 ± 102	1001 ± 478
	Min	0	1	7	140	240	675
	Max	0.83	10	8	496	680	2780
Boukan	Mean	0.31 ± 0.15	3.61 ± 3.5	7.23 ± 0.18	273.6 ± 92	326.3 ± 125	710.6 ± 259.8
	Min	0.02	1	7	100	24	274
	Max	0.57	20	8	488	620	1423
Shahin Dezh	Mean	0.28 ± 0.1	3.31 ± 1.1	7.28 ± 0.11	221.52 ± 51.6	300.5 ± 73.3	568.7 ± 181
	Min	0.13	2	7	104	148	275
	Max	0.54	6	7	316	468	1087
Maku	Mean	0.73 ± 0.67	0.89	7.4 ± 0.25	345.5 ± 154.6	367 ± 132	1096.4 ± 671
	Min	0	0	7	88	96	233
	Max	3.45	4	8	628	656	3270
Piranshahr	Mean	0.094 ± 11	4.29 ± 3.2	7.53 ± 0.17	177.8 ± 56.8	195 ± 62	407.5 ± 82
	Min	0	2	7	0	22	267
	Max	0.4	13	8	236	288	591
Total	Mean	0.32 ± 0.37	3.58 ± 11.7	7.39 ± 0.23	283.97 ± 117	327.2 ± 136	810.8 ± 527
	Min	0	0	7	0	22	82
	Max	3.45	218	8	720	912	3360

**Table 3:** Correlation (Pearson correlation) between fluoride and different water quality parameters in West Azerbaijan province.

	F	EC	pH	TH	Ca	Mg	Na	K	ALK	NO_3_	SO_4_
F	1	0.415**	0.02	0.304**	0.112	0.287**	0.557**	0.467**	0.462**	0.007	0.409**
EC	0.415**	1	−0.084	0.843**	0.52**	0.6**	0.912**	0.528**	0.61**	−0.02	0.803**
pH	0.02	−0.084	1	−0.176**	−0.247**	−0.036	−0.059	−0.043	−0.211**	0.028	0.046
TH	0.304**	0.843**	−0.176**	1	0.662**	0.68**	0.704**	0.382**	0.667**	−0.07	0.667**
Ca	0.112	0.52**	−0.247**	0.662**	1	0.221**	0.344**	0.13*	0.41**	−0.076	0.365**
Mg	0.287**	0.6**	−0.036	0.68**	0.221**	1	0.552**	0.293**	0.492**	−0.041	0.486**
Na	0.557**	0.912**	−0.059	0.704**	0.344**	0.552**	1	0.607**	0.602**	0.007	0.715**
K	0.467**	0.528**	−0.043	0.382**	0.13*	0.293**	0.607**	1	0.457**	−0.014	0.37**
ALK	0.462**	0.61**	−0.211**	0.667**	0.41**	0.492**	0.602**	0.457**	1	−0.092	0.283**
NO_3_	0.007	−0.02	0.028	−0.07	−0.076	−0.041	0.007	−0.014	−0.092	1	0.004
SO_4_	0.409**	0.803**	0.046	0.667**	0.365**	0.486**	0.715**	0.37**	0.283**	0.004	1

** Correlation is significant at the 0.01 level (2-tailed).

* Correlation is significant at the 0.05 level (2-tailed).

## 2 Experimental design, materials and methods

### 2.1 Description of study area

**Fig. 1:**
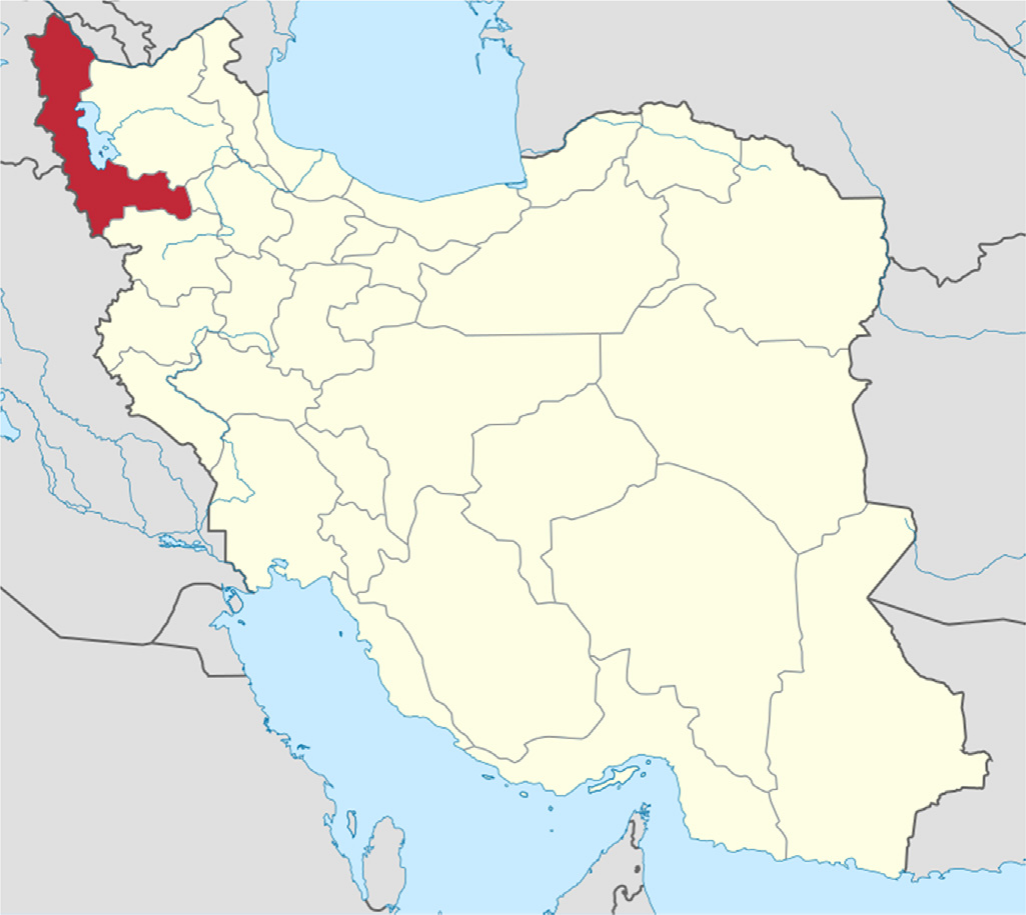
Study area.

West Azerbaijan province is one of the 31 provinces of Iran (Fig. 1). It is in the northwest of the country in coordination  37.5528°N 45.0759°E [1], [2], [3], [4].

### 2.2 Sample collection and analytical procedures

This research is a descriptive-applied study. The studied subject was drinking water resources in the cities of West Azerbaijan province. In order to carry out this study, the results of chemical analysis of urban and rural water resources of 13 cities of West Azerbaijan province were collected as a sample in 2017. In the following, data on the concentration of cations and anions, hardness, electrical conductivity, turbidity, TDS, and alkalinity were extracted and the values of mean, standard deviation and correlation coefficient were calculated. It should be noted that all of mentioned parameters were measured according to the standard Methods for the Examination of Water and Wastewater [5], [6], [7], [8], [9], [10], [11], [12], [13], [14], [15]. This province is limited from the north to Azerbaijan and Turkey, from the west to Turkey and Iraq, from the east to the provinces of East Azerbaijan and Zanjan, and south to the Kurdistan province. The province׳s area is 37,059 km^2^, that is the 13th largest province in the country in terms of area. According to the 2006 census, the population of the province is 2,873,459 people and has 17 cities [1], [16], [17], [18], [19], [20], [21], [22], [23].
